# The relationship between patterns of artificial intelligence use, academic resilience, and burnout among graduate students in special education departments at Saudi universities

**DOI:** 10.3389/fpsyg.2026.1776966

**Published:** 2026-05-04

**Authors:** Reda Ebrahim Mohamed Elashram, Liyla Marzouk Alamri

**Affiliations:** Department of Special Education, College of Education, Imam Mohammad Ibn Saud Islamic University (IMSIU), Riyadh, Saudi Arabia

**Keywords:** academic resilience, artificial intelligence applications, burnout, graduate students, Saudi universities, special education

## Abstract

**Introduction:**

Higher education institutions face increasing challenges in maintaining the psychological well-being of graduate students amid intensive academic pressures and rapid digital transformation. This study investigated the relationships between patterns of artificial intelligence (AI) application use, academic resilience, and burnout among graduate students in special education departments at Saudi universities, and determined the predictive capacity of these variables for burnout.

**Methods:**

A cross-sectional correlational descriptive design was employed. Data were collected from 367 graduate students (207 males, 160 females) using the Maslach Burnout Inventory (MBI-SS), the Brief Resilience Scale (BRS), and a developed scale for AI application usage patterns.

**Results:**

Results revealed low levels of AI application use and academic resilience, in contrast to high levels of burnout. Significant negative correlations were found between AI usage patterns and burtenout (*r* = −0.541, *p* < 0.001), and between academic resilience and burnout (*r* = −0.437, *p* < 0.001). AI application usage patterns explained 34.1% of the variance in burnout (*R*^2^ = 0.341, *f*^2^ = 0.52, a large effect size), while academic resilience explained 19.1% (*R*^2^ = 0.191, *f*^2^ = 0.24, medium effect).

**Discussion:**

These findings highlight the potential of technological competence as a psychological resource associated with reduced burnout. Structured AI training programs, institutional resilience interventions, and optimized research workloads are recommended in alignment with Saudi Vision 2030.

## 1 Introduction

Higher education institutions are a cornerstone in building national capabilities and developing advanced human capital. The inherently deep research nature of graduate programs imposes complex cognitive, skill-based, and emotional demands on their students, necessitating adaptive capacities and supportive psychological and institutional resources (Alshobaili et al., 2021). In various contexts, universities face mounting challenges in preserving students’ psychological well-being, especially with the increasing academic pressures and research expectations associated with graduate-level studies.

Burnout is a widespread phenomenon with substantial negative impacts, defined as a multidimensional psychological syndrome characterized by emotional exhaustion (depletion of emotional resources), depersonalization (loss of connection and engagement), and a diminished sense of personal accomplishment (a reduced sense of competence and effectiveness) (Alqifari et al., 2021; Maslach et al., 2001). It is a state resulting from chronic exposure to stressors that have not been successfully managed (Alsaad et al., 2021). Burnout has been shown to negatively affect academic and professional performance and increase the likelihood of withdrawal from the educational path (Alsharif, 2020; Alsaad et al., 2021). While burnout originally emerged as an occupational syndrome, academic burnout represents its manifestation within educational settings. In this context, burnout encompasses emotional exhaustion, depersonalization toward academic tasks, and a diminished sense of academic accomplishment (Schaufeli et al., 2002), distinguishing it from broader occupational conceptualizations.

In contrast, academic resilience serves as a fundamental protective factor, referring to a student’s ability to recover quickly and adapt positively to difficult circumstances and academic challenges (Al-Zain and Abdulsalam, 2022). This capacity is considered a critical indicator of psychological health and long-term success. Research has demonstrated that students with higher resilience are less susceptible to emotional exhaustion (Alsharif, 2020), underscoring its protective role in shielding students from the negative consequences of a high-pressure academic environment (Alsharif et al., 2023).

These academic pressures are concurrent with a fundamental transformation in learning and research methods, as reliance on artificial intelligence (AI) applications in the academic sphere grows (Almankory, 2025). The patterns of use of these applications by graduate students—in content creation, research idea formulation, and literature summarization (Alazemi et al., 2024; Cheng et al., 2025)—have become a new and influential factor that warrants investigation. These technologies offer opportunities to simplify research tasks and increase efficiency (Alotaibi, 2025), but their unregulated use can lead to adverse outcomes, such as weakening critical thinking, fostering over-dependence, or raising academic integrity issues that exacerbate burnout (Hurshman et al., 2025).

The study of this interplay between patterns of technological use, academic resilience, and burnout is of utmost importance in the Kingdom of Saudi Arabia, which has established an ambitious national framework for digital transformation under its Vision 2030 goals (Alotaibi and Alshehri, 2023). This vision necessitates the integration of advanced technologies in higher education to enhance the quality of outcomes, which requires a thorough assessment of how this integration impacts students’ psychological well-being (Alruwaili et al., 2025).

The social and cultural context in Saudi Arabia, with its high familial and societal expectations for the achievements of graduate students, adds an additional layer of pressure that may contribute to elevated rates of burnout among some students (Al-Awad, 2024; Alshobaili et al., 2021). Local studies in the educational and medical sectors—fields that share similar pressure characteristics with special education programs—also show a noticeable increase in burnout rates (Altharman et al., 2023; Siraj et al., 2023).

Despite this importance, three distinct research gaps limit our understanding of this phenomenon: First, the majority of Saudi research focuses on undergraduate students in health specializations (Almurdi et al., 2024; Siraj et al., 2023), with a clear neglect of graduate students in the humanities and educational disciplines, including special education. Second, findings regarding the role of resilience as a protective factor against burnout have been inconsistent; while some studies have found a significant negative correlation (Altharman et al., 2023), others have found no significant difference (Almurdi et al., 2024). Third, studies directly linking patterns of technology use to both academic resilience and burnout within an integrated model are scarce, particularly in a context that emphasizes the quality of research outputs in graduate studies.

Therefore, the present study seeks to answer the research questions by determining the levels of these variables, exploring their correlational relationships, and examining the predictive capacity of AI application usage patterns and resilience on burnout. To our knowledge, no prior study has examined AI usage patterns, academic resilience, and burnout within a single predictive model targeting graduate students in special education departments in Saudi Arabia, constituting a substantive contribution to the literature at the intersection of educational technology and student well-being. It is anticipated that this work will enrich the scientific field by providing a comprehensive analytical framework that connects technological factors with psychological and academic variables in a specific cultural and institutional context, supporting student well-being and the sustainability of high-quality higher education outcomes.

## 2 Theoretical and conceptual framework and literature review

### 2.1 Theoretical perspectives

The current study is grounded in a set of theoretical perspectives that explain the complex interaction between its variables. According to the World Health Organization’s classification, burnout is viewed as a syndrome resulting from chronic stress in an academic context that has not been successfully managed (Alsaad et al., 2021; Maslach et al., 2001). Burnout is traditionally described through three primary dimensions: emotional exhaustion, depersonalization, and a reduced sense of personal accomplishment (Alsharif, 2020; Altharman et al., 2023).

One of the most prominent frameworks for explaining this phenomenon is the Job Demands-Resources (JD-R) model, which posits that burnout arises when job demands (such as academic pressures and research workload) exceed a student’s available resources, leading to the depletion of emotional energy (Asfahani, 2024; Siraj et al., 2022). In contrast, academic resilience represents a key protective resource within this model (Al-Awad, 2024). Resilience is defined as the ability to recover, adapt positively, and persevere despite facing academic challenges (Abood and Hmaid, 2023; Al-Zain and Abdulsalam, 2022). Literature has confirmed that resilience is not merely an inherited trait but a dynamic process that can be learned and enhanced, acting as a powerful protective factor against emotional exhaustion and burnout (Alsharif, 2020; Wadi et al., 2024).

**Figure 1:**
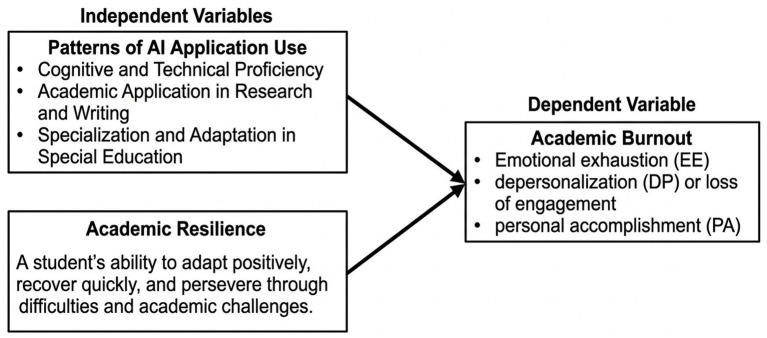
Conceptual model linking Al usage, academic resilience, and academic burnout, arrows indicate the direction of proposed associations.

In light of the digital transformation, theoretical perspectives must incorporate patterns of AI application use into the equation of demands and resources. Advanced language modeling applications can be considered a vital new resource, as they support research efficiency, facilitate the formulation of ideas, and assist in summarizing literature, thereby increasing a student’s sense of accomplishment (Alotaibi, 2025; Alazemi et al., 2024). This support helps reduce the cognitive and time-related burden, which in turn enhances academic resilience (Abdelrahman et al., 2025). The theoretical mechanism linking AI application usage patterns to reduced burnout is grounded in cognitive offloading; by delegating repetitive and time-consuming tasks (e.g., literature summarization) to AI, students free up cognitive resources. This reduction in workload demands directly mitigates emotional exhaustion. Conversely, disorganized use of these applications represents a new threat and pressure, such as causing excessive digital dependency or creating ethical pressures related to academic integrity (Aldawsari and Almohish, 2024; Cheng et al., 2025). Therefore, determining whether AI application usage patterns function as a resource to enhance resilience or as a stressor that increases burnout is the theoretical foundation for answering this study’s research questions (see Figure 1).

### 2.2 Patterns of AI application use among graduate students in special education at Saudi universities

Process at the graduate level, which requires high levels of cognitive and research engagement (Almankory, 2025; Alotaibi, 2025). The rapid developments in Generative AI tools have led to a profound shift in how students access information and accomplish their academic tasks (Alazemi et al., 2024; Cheng et al., 2025). Patterns of using these applications refer to their structured use in specific academic tasks. This includes improving research writing, formulating ideas, and summarizing literature. Furthermore, they provide immediate feedback mechanisms that facilitate deep academic engagement.

In this study, AI application usage patterns are operationally defined as the frequency, purpose, and proficiency with which graduate students employ generative AI tools within three domains: cognitive and technical engagement, academic and research application, and specialization-specific adaptation in special education contexts.

In the local context, the adoption of AI applications has become a strategic necessity for Saudi universities, aligning with the Vision 2030 goals for digital transformation and the development of higher education (Alotaibi and Alshehri, 2023; Khan et al., 2025). The widespread use of these tools among students indicates their acceptance as part of the contemporary academic environment (Alazemi et al., 2024; Alotaibi, 2025). This acceptance is further shaped by users’ readiness and the presence of institutional support structures that facilitate the meaningful adoption of generative AI in higher education (Sallam et al., 2025). These tools offer significant opportunities for graduate students in special education by enabling them to access information and educational resources continuously, which enhances personalized learning opportunities and increases the efficiency of the research process (Aldawsari and Almohish, 2024; Almankory, 2025). These systems can contribute to alleviating routine research burdens, such as organizing references or generating basic text, which increases students’ sense of effectiveness and accomplishment (Cheng et al., 2025).

Cultural dimensions significantly moderate AI adoption in Saudi higher education, manifesting as a dualistic tension between national ambition and traditional values. While Vision 2030’s digital transformation goals catalyze widespread institutional enthusiasm (Alotaibi and Alshehri, 2023), deep-seated concerns regarding academic authenticity and digital dependency foster significant ambivalence (Aldawsari and Almohish, 2024). This socio-cultural dynamic, characterized by a commitment to academic integrity, serves as a critical moderator within regional Technology Acceptance Model (TAM) frameworks, where the pursuit of innovation intersects with culturally rooted pedagogical standards (Alotaibi and Alshehri, 2023; Aldawsari and Almohish, 2024).

However, this expansion in use is not without its fundamental challenges and risks. The primary risk lies in the threat to academic integrity, as these tools can be used to generate complete research drafts, raising questions about the authenticity of the work and the true contribution of the researcher (Hurshman et al., 2025). There is also the prominent issue of excessive digital dependency, which may lead to a decline in students’ critical thinking and independent analytical skills (Aldawsari and Almohish, 2024). Furthermore, the academic community faces challenges related to the need to distinguish between structured use that supports learning and unstructured use that may exacerbate ethical and professional issues (Alotaibi and Alshehri, 2023; Cheng et al., 2025). This study seeks to analyze these usage patterns in relation to academic resilience and burnout, with the aim of identifying factors that enhance academic well-being.

### 2.3 Academic resilience among graduate students in special education at Saudi universities

Academic resilience is a core concept in the context of advanced higher education, representing students’ ability to persevere, adapt positively, and achieve good outcomes despite academic challenges and psychological pressures (Altharman et al., 2023; Cassidy, 2016). Resilience is generally defined as the psychological mechanism that enables an individual to recover and adapt successfully to stress and adversity (Alsharif, 2020; Al-Zain and Abdulsalam, 2022). The literature has confirmed that this characteristic is not merely a fixed, innate trait but a dynamic process that can be developed and enhanced, playing a crucial role in maintaining overall psychological well-being (Abdelrahman et al., 2025; Wadi et al., 2024). The dimensions of resilience are manifested in students’ ability to regulate their emotions, feel a sense of autonomy and hope, and develop positive thinking mechanisms for problem-solving (Abood and Hmaid, 2023; Al-Awad, 2024).

The majority of studies agree that academic resilience acts as a primary protective factor against burnout (Alsharif et al., 2023). Regional research using reliable measures such as the Brief Resilience Scale (BRS) and the Connor-Davidson Resilience Scale (CD-RISC-10) has shown a strong and negative correlation between high levels of resilience and reduced symptoms of burnout (Al-Awad, 2024; Alsharif, 2020; Almurdi et al., 2024; Siraj et al., 2023). For example, one Saudi study revealed that resilience was a key protective factor against emotional exhaustion among students in health specializations (Alsharif, 2020), while another study found that resilience was negatively correlated with depersonalization and positively with personal accomplishment (Siraj et al., 2022).

The evidence shows that Falah et al. (2025) found a significant negative relationship between burnout and psychological resilience among psychology and educational sciences students. Similarly, Gianjacomo et al. (2025) demonstrated that resilience had a significantly negative effect on academic burnout among undergraduate students, with resilience mediating approximately 54.9% of the relationship between social support and burnout.

This protective function has also been documented in non-academic Saudi contexts; for instance, studies examining resilience among healthcare workers during crises (e.g., AlAteeq et al., 2020) reported comparable buffering mechanisms, suggesting that resilience operates as a trans-contextual resource within the Saudi socio-cultural milieu.

However, the literature also contains debates about the extent of resilience’s impact as a sole predictor. Some findings have shown that resilience was not a statistically significant predictor of burnout after introducing other variables into the regression model (Al-Zain and Abdulsalam, 2022). As for the reported levels in the Saudi context, they are varied; resilience scores have ranged from low to moderate among different samples of health profession students (Al-Zain and Abdulsalam, 2022; Almurdi et al., 2024). A review reveals a clear research gap, as the majority of local studies have focused on undergraduate students in medical specializations, with a noticeable neglect of graduate students in educational and humanities disciplines. The contribution of these studies is often limited to cross-sectional designs, which restricts the ability to infer causal relationships or track the development of resilience over time.

### 2.4 Burnout among graduate students in special education at Saudi universities

Burnout is a complex, multidimensional phenomenon, included in the International Classification of Diseases, 11th Revision (ICD-11) as a syndrome resulting from chronic workplace or academic stress that has not been effectively managed (Alsaad et al., 2021; Maslach et al., 2001). The academic and professional concept revolves around three core dimensions: emotional exhaustion, depersonalization or loss of engagement, and a reduced sense of personal accomplishment (Alsharif, 2020). Research has shown that emotional exhaustion, in particular, is the most prevalent and analytically studied dimension, representing a state of physical and emotional energy depletion due to excessive psychological demands (Al-Awad, 2024; Siraj et al., 2023).

In the Kingdom of Saudi Arabia, studies that have adopted a cross-sectional correlational design and used the Maslach Burnout Inventory (MBI) agree on the existence of high prevalence rates of burnout, especially among students in health specializations (Alqifari et al., 2021; Dabbagh et al., 2023). These rates have varied widely, from low to high proportions exceeding 75% in some samples of medical trainees (Dabbagh et al., 2023). The collective findings indicate that burnout is associated with academic factors such as high academic pressure and progression through the years of study (Al-Awad, 2024; Siraj et al., 2023), while there is variation and research debate regarding the role of other demographic factors.

One of the most debated points is the relationship between gender and burnout. Several systematic reviews have shown that females are more prone to emotional exhaustion and higher levels of burnout compared to males (Alqifari et al., 2021; Alshobaili et al., 2021). However, other studies have concluded that there are no statistically significant differences in overall burnout levels between genders (Alsaad et al., 2021; Alsharif et al., 2023). As for protective factors, there is a strong consensus that academic resilience plays a powerful negative role in countering burnout, acting as a buffer that reduces emotional exhaustion and helps individuals recover from stress (Alsharif, 2020; Al-Zain and Abdulsalam, 2022).

Despite the extensive research, a significant gap remains, as the vast majority of local studies target undergraduate students and clinical trainees in medical fields (Alsharif, 2020; Dabbagh et al., 2023), leaving a deficit in understanding the dynamics of burnout among graduate students, especially in non-health disciplines like special education (Alkhunini and Alsawalem, 2025). The scientific need calls for the use of current methodologies to assess this phenomenon in this specific context, considering that the nature of graduate studies may differ from the pressures of clinical training.

### 2.5 AI application usage patterns and academic resilience as predictors of burnout among graduate students in special education at Saudi universities

The core of the current study rests on testing an analytical model aimed at determining the relative impact of two main variables—academic resilience and patterns of smart application use—in predicting the phenomenon of burnout among graduate students. This approach is essential for deepening the understanding of the dynamics of psychological well-being in a changing academic environment (Al-Zain and Abdulsalam, 2022).

Studies that have employed a correlational survey design and used reliable measurement tools agree that academic resilience constitutes a strong protective factor against burnout (Alsharif, 2020; Altharman et al., 2023). Regression analyses have revealed a significant and negative relationship; as resilience increased, levels of emotional exhaustion decreased (Alsharif et al., 2023; Alsharif, 2020). In the context of predictive models, resilience demonstrated its ability to significantly reduce the likelihood of burnout, confirming its role as a crucial internal resource for coping with stress (Al-Awad, 2024). However, some statistical findings show that the role of resilience may weaken as a sole predictor of burnout in models that include strong stressor variables, suggesting the need to add other contextual variables (Alsharif et al., 2023; Al-Zain and Abdulsalam, 2022). Within the JD-R framework, AI tools are theorized to reduce burnout through three interrelated mechanisms: (1) cognitive offloading—by automating routine research tasks, they reduce cognitive load and emotional depletion; (2) self-efficacy enhancement—successful AI-assisted task completion reinforces students’ sense of competence; and (3) temporal resource liberation—time saved through AI assistance can be reallocated to higher-order analytical activities, ultimately moderating the demand-resource imbalance.

The inclusion of the variable “patterns of AI application use” in a burnout prediction model represents a clear research gap in the current literature, especially when targeting graduate students (Alotaibi and Alshehri, 2023). Theoretically, these applications can act as a supportive resource, offering mechanisms to simplify complex research tasks, such as summarizing literature or formulating ideas, which increases students’ sense of personal accomplishment and reduces exhaustion (Alotaibi, 2025). Conversely, unregulated use or excessive digital reliance can be a source of stress, threatening the erosion of analytical skills and leading to an increase in burnout levels. The digital overload resulting from the increased demand to stay connected can also contribute to exacerbating emotional exhaustion.

The deficiency lies in the absence of studies applying logistic or multiple regression analysis to test these tripartite relationships in an integrated model, especially within the graduate student population in special education. The current cross-sectional methodology gains its importance from its ability to provide a “snapshot” of these contemporary relationships, with the caveat that results must be interpreted cautiously due to its inability to establish direct causality. Therefore, this study seeks to bridge this gap through a predictive model that determines the relative and ranked importance of academic resilience and AI application usage patterns in explaining the variance in burnout.

## 3 Research problem and questions

The academic environment of graduate studies, especially in disciplines with high pressure and professional responsibilities such as special education, is a fertile ground for the development of intense psychological stress (Alsaad et al., 2021; Altharman et al., 2023). These chronic pressures, if not managed effectively, lead to the burnout syndrome (Al-Awad, 2024; Maslach et al., 2001). Systematic reviews indicate that burnout rates among students in health and educational specializations in the Kingdom are high and warrant intervention (Alshobaili et al., 2021; Dabbagh et al., 2023). Academic resilience emerges as a key protective factor, and a strong inverse relationship between it and emotional exhaustion and burnout has been established (Alsharif, 2020; Altharman et al., 2023).

In the context of the national drive towards digital transformation and the implementation of Saudi Vision 2030 (Alotaibi and Alshehri, 2023; Khan et al., 2025), the integration of AI applications in research and advanced learning has accelerated (Almankory, 2025). These applications offer immense potential for simplifying complex research tasks (Alazemi et al., 2024; Cheng et al., 2025). However, researchers warn that excessive or unethical use poses a serious threat to academic integrity and critical thinking, which may lead to cognitive overload and increase the risk of burnout (Alotaibi, 2025; Aldawsari and Almohish, 2024; Hurshman et al., 2025).

The need for this study stems from three main research gaps: First, despite local interest in the phenomenon of burnout, most studies still target students in medical and health specializations at the undergraduate level (Dabbagh et al., 2023; Siraj et al., 2023), with a near-complete neglect of graduate students, especially in specific disciplines like special education. Second, studies related to AI applications in the Saudi context have so far focused on the opportunities and challenges associated with institutional adoption or evaluating content quality (Alotaibi and Alshehri, 2023; Aldawsari and Almohish, 2024), without directly linking them to key psychological variables: resilience and burnout. Third, there is a need for an integrated model that tests the interaction among these three variables in a social and cultural context that adds unique pressures, especially on female students (Al-Awad, 2024; Alshobaili et al., 2021). Determining whether patterns of using these applications act as a supportive factor for resilience or as a catalyst for burnout is a pressing necessity for supporting academic well-being and ensuring the quality of research outcomes in Saudi universities.

### 3.1 Research questions

Based on the research problem and gaps, the current study seeks to answer the following questions:

1. What are the levels of AI application usage patterns, academic resilience, and burnout among graduate students in special education departments at Saudi universities?
2. Is there a statistically significant correlational relationship between patterns of AI application use, academic resilience, and burnout among graduate students in special education departments at Saudi universities?
3. Are patterns of AI application use and academic resilience statistically significant predictors of burnout among graduate students in special education departments at Saudi universities?

### 3.2 Hypotheses

*H1*: There is a statistically significant negative relationship between AI application usage patterns and academic burnout among graduate students in special education departments.

*H2*: Academic resilience is a statistically significant negative predictor of burnout.

*H3*: Patterns of AI application use and academic resilience statistically and significantly predict burnout.

## 4 Materials and methods

### 4.1 Method and design

The current study employed a descriptive correlational cross-sectional design. This design is suitable for describing the phenomenon as it occurs in reality and for uncovering the correlational relationships between the studied variables (Creswell and Creswell, 2023). In this predictive design, patterns of AI application use and academic resilience served as the independent (predictor) variables, while burnout was designated as the dependent (criterion) variable. This design is considered appropriate for the research objectives, which aim to determine the levels of AI application usage patterns, academic resilience, and burnout, in addition to exploring the ability of the independent variables to predict burnout (Creswell and Plano Clark, 2018).

Data were collected using a self-administered electronic questionnaire. This method is common and effective in research that deals with psychological and behavioral variables in higher education institutions (Alqifari et al., 2021; Alsharif, 2020; Siraj et al., 2022).

## 5 Participants

### 5.1 Population and sample

The study population consisted of all graduate students (Master’s and Ph.D.) registered in special education departments at Saudi universities, totaling 595 students according to official statistics. To obtain a representative sample of the population, a convenience sampling method was used. This method is appropriate in situations where it is difficult to provide a comprehensive and up-to-date list of all individuals in the population (Sykes et al., 2018).

This method was chosen due to the lack of a precise sampling frame covering all graduate students in special education departments across Saudi universities, in addition to the temporal and procedural constraints associated with using probability sampling. Despite this, the researchers ensured a degree of geographical (covering six universities from different regions) and specialization (four tracks in special education) diversity. It is acknowledged that this type of sampling may limit the potential for statistical generalization of the results to the entire population, and this issue will be discussed in the limitations section.

A convenience sampling procedure was executed by distributing the survey link through official departmental email lists, graduate students’ WhatsApp groups, and direct academic channels facilitated by faculty members across the six participating universities. No incentives were offered for participation. The electronic questionnaire was distributed via the Google Forms platform to graduate students in special education departments at six public Saudi universities (King Saud University, University of Tabuk, Prince Sattam bin Abdulaziz University, King Abdulaziz University, King Khalid University, and University of Ha’il) during the period from October 23 to December 2, 2025, of the first semester.

The minimum required sample size was determined using Cochran’s formula for calculating sample size from a finite population, at a 95% confidence level and a 5% margin of error (Thompson, 2012), which resulted in a target sample size of 234 participants. The actual sample size (367) exceeded the minimum requirement by 57%, providing high statistical power (Power >0.90) to detect medium effects (*f*^2^ = 0.15) in multiple regression analysis (Cohen, 1988).

A total of 423 participants responded to the questionnaire (initial response rate = 71.09%). After examining the responses, 56 responses were excluded for systematic reasons (32 incomplete responses with over 20% missing data, and 24 responses showing a repetitive pattern indicating a lack of seriousness). These 56 excluded responses were designated as the sample for calculating the psychometric properties of the instruments, with ages ranging from 24 to 35 years (mean age = 30.21, SD = 1.11). The remaining 367 responses constituted the main sample for the primary statistical analyses, with ages ranging from 24 to 35 years (mean age = 31.22, SD = 1.23).

### 5.2 Inclusion and exclusion criteria

Inclusion criteria included being currently enrolled in a graduate program (Master’s or Ph.D.) in special education departments at Saudi universities. Exclusion criteria involved excluding students not enrolled in graduate programs (such as undergraduate students). Additionally, students who reported a history of serious psychological disorders (such as depression and severe anxiety) or who were currently receiving psychological treatment that might affect their responses to the psychological scales were excluded to ensure the accuracy of the data (see Table 1).

**Table 1:** Descriptive statistics of participants in the main study by variable.

Variable	Categories	*N*	Percentage
Gender	Male	207	56.40%
	Female	160	43.59%
Academic level	Master’s	219	59.67%
	Doctorate	148	40.32%
Specialization track in special education	Specific learning disorder	103	28.06%
	Intellectual disability	86	23.43%
	Autism spectrum disorder	84	22.88%
	Giftedness and creativity	94	25.61%
Total		367	

## 6 Measures

The study instrument included a set of scales to collect data on the three variables:

### 6.1 Burnout

The Maslach Burnout Inventory (MBI), considered the gold standard for measuring burnout (Maslach et al., 2008; Maslach et al., 1997), was used. In this study, the abbreviated version for students (MBI-SS), developed by Schaufeli et al. (2002) to suit the academic environment, was utilized. It was then condensed into a practical 10-item format that retains the original three-factor structure: emotional exhaustion (EE) (4 items), depersonalization (DP) or loss of engagement (3 items), and personal accomplishment (PA) (3 items).

A 7-point Likert scale ranging from 0 (*Never*) to 6 (*Every day*) is used. High scores on the exhaustion and depersonalization dimensions, and a low score on the personal accomplishment dimension, are interpreted as indicators of a high level of academic burnout.

This study used the Arabic version adapted for the Saudi environment, which has been previously used and validated for its psychometric properties in several local studies on university students (Alsharif, 2020; Siraj et al., 2022, 2023). The researchers conducted a careful linguistic review to ensure the wording was appropriate for the context of graduate students in special education, with minor modifications made to two items for clarity without altering the original meaning.

The original version of the MBI-SS has demonstrated excellent reliability and validity coefficients across various cultures. In the Saudi context specifically, previous studies have shown acceptable to good reliability coefficients: Alsharif (2020) on dental students: *α* = 0.78–0.84; Siraj et al. (2022) on respiratory therapy students: α = 0.74–0.81; and Siraj et al. (2023) on health professionals: α = 0.76–0.85. Furthermore, confirmatory factor analysis studies have confirmed a good fit for the three-factor structure in the Arab context (CFI > 0.90, RMSEA < 0.08).

### 6.2 Academic resilience

The Brief Resilience Scale (BRS), developed by Smith et al. (2008) at the University of Colorado, was used. It consists of 6 items that alternate between positive (items 1, 3, 5) and negative (items 2, 4, 6) phrasing to mitigate response bias (Rodríguez-Rey et al., 2016). It measures a student’s ability to adapt positively, recover quickly, and persevere through difficulties and academic challenges. Responses are formatted on a 5-point Likert scale, where higher scores indicate greater resilience.

The BRS has demonstrated robust psychometric properties across diverse cultural contexts. In the international context, the original study reported high internal consistency coefficients ranging from 0.80 to 0.91 across four different samples (Smith et al., 2008), and subsequent studies, such as the Spanish version which recorded strong reliability (*α* = 0.83, *ω* = 0.84; Rodríguez-Rey et al., 2016), have supported these findings. A systematic review of 149 studies showed an average reliability of 0.81 (Allan, 2021). Locally, in the Saudi context, the scale has shown good stability and reliability, with alpha-Cronbach values in previous studies ranging from 0.76 among dental students (Al-Zain and Abdulsalam, 2022), to 0.78 among respiratory therapy students (Siraj et al., 2022), up to 0.81 among health professionals (Siraj et al., 2023).

### 6.3 Psychometric properties of the two scales

Arabic versions of both instruments have demonstrated acceptable psychometric properties in prior Saudi studies: the MBI-SS showed adequate factorial validity in samples of Saudi health profession students (Alsharif, 2020; Siraj et al., 2022), and the BRS Arabic version demonstrated configural equivalence with the original scale (Al-Zain and Abdulsalam, 2022). The present study adopted these validated versions after a committee review to ensure contextual appropriateness for special education graduate students.

The burnout and academic resilience scales were presented to a committee of five expert reviewers specializing in measurement, evaluation, and educational psychology. The agreement rates among the reviewers were 92.5 and 96.7%, respectively, with minor comments related to the phrasing of one item on the resilience scale. The pilot sample results (*n* = 56) showed strong indicators of the scales’ suitable psychometric properties. Item-total correlations were statistically significant, ranging from 0.457 to 0.797 for the burnout scale and from 0.632 to 0.711 for the resilience scale, reflecting good construct validity and internal consistency. The reliability coefficients also exceeded acceptable limits; the burnout scale recorded a Cronbach’s alpha of 0.766 and an omega of 0.752, while the resilience scale’s values were *α* = 0.787 and *ω* = 0.773. These indicators demonstrate that both scales possess high levels of accuracy and reliability, reinforcing their suitability for use in the context of the current study.

### 6.4 AI application usage patterns scale (developed for the current study)

Due to the lack of reliable Arabic scales that measure the usage patterns of generative AI applications by graduate students in academic and research contexts, the researchers developed a new scale tailored to the objectives of the current study and the Saudi cultural context. The researchers reviewed specialized literature on the use of AI applications in higher education, drawing specifically on studies by Alotaibi (2025), Alazemi et al. (2024), Akbar (2025), and Cheng et al. (2025), and extracted the main conceptual dimensions of usage patterns.

Based on the extracted conceptual framework, the researchers formulated an initial 28 items covering three main dimensions: Cognitive and Technical Proficiency (9 items), Academic Application in Research and Writing (10 items), and Specialization and Adaptation in Special Education (9 items). A five-point Likert scale was used (1 = Does not apply at all, 5 = Applies to a very large extent).

### 6.5 Validity evidence

#### 6.5.1 Content Validity

The initial version of the scale was presented to seven expert reviewers (three in educational technology, three in measurement and evaluation, and one in special education) from different Saudi universities, all holding doctoral degrees with at least 5 years of experience in higher education. They were asked to evaluate the clarity of the wording, the relevance of the items to the intended dimension, and cultural appropriateness. The agreement rate among the reviewers was 85.7%. Based on their comments, 6 items were rephrased, two similar items were merged, and two items considered outside the scope were deleted, resulting in a semi-final version of the scale with 24 items.

The scale was administered to a pilot sample of 56 students from the same population (but outside the main sample) to examine its initial psychometric properties. Item-subscale correlations were calculated, and 4 items with weak correlations (*r* < 0.40) were excluded. This stage resulted in the final version of the scale, consisting of 20 items distributed across three dimensions: Cognitive and Technical Proficiency (7 items), Academic Application in Research and Writing (7 items), and Specialization and Adaptation in Special Education (6 items).

#### 6.5.2 Construct validity

Pearson correlation coefficients were calculated between the score of each item and the total score of the dimension to which it belongs (after excluding the item’s score from the dimension’s total). The correlation coefficients ranged from 0.445 to 0.882, all of which were statistically significant at the 0.01 level, supporting the internal consistency of the scale.

### 6.6 Reliability evidence

The reliability of the scale was calculated using two methods: Cronbach’s alpha (*α*) and McDonald’s omega (*ω*), the latter being more accurate when the assumption of equal factor loadings is not met (Dunn et al., 2014). The results showed acceptable to good reliability values for all dimensions (see Table 2): Cognitive and Technical Proficiency (*α* = 0.835, *ω* = 0.825), Academic and Research Application (*α* = 0.786, *ω* = 0.791), and Specialization and Adaptation (*α* = 0.852, *ω* = 0.861), indicating suitable internal consistency for research use.

**Table 2:** Summary of psychometric properties for the study’s scales.

Scale/dimension	No. of items	Item-dimension correlation range	Cronbach’s *α*	McDonald’s *ω*
AI application usage patterns scale				
Cognitive and technical proficiency	7	0.648–0.867	0.835	0.825
Academic and research application	7	0.445–0.787	0.786	0.791
Specialization and adaptation	6	0.505–0.882	0.852	0.861
Academic resilience scale	6	0.632–0.711	0.787	0.773
Burnout scale				
Emotional exhaustion	4	0.457–0.776	0.740	0.743
Depersonalization/loss of engagement	3	0.642–0.797	0.738	0.740
Personal accomplishment (low)	3	0.610–0.735	0.731	0.736
Total burnout score	10	–	0.766	0.752

Psychometric properties were calculated based on the pilot sample data (*n* = 56).

The results in Table 2 indicate that all reliability coefficients (Cronbach’s alpha and McDonald’s omega) exceed the acceptable minimum of 0.700 set by Nunnally and Bernstein (1994), which indicates excellent reliability for the used scales. The item-dimension correlations were all statistically significant at the 0.01 level, which enhances the construct validity of the instruments.

## 7 Statistical methods

Data were analyzed using SPSS version 29. Descriptive statistics (means, standard deviations, and percentages) were used to describe the sample characteristics and variable levels. Cronbach’s alpha and McDonald’s omega coefficients were calculated to examine the reliability of the instruments, and item-dimension correlations were calculated to verify construct validity. To examine the relationships between variables, Pearson’s correlation coefficient was applied. To check the predictive ability, standard multiple regression and simple linear regression analyses were used. Each research question is addressed through a corresponding statistical procedure: Question 1 employs descriptive statistics (*M*, SD, and level classification); Question 2 employs Pearson’s correlation; and Question 3 employs simple and multiple linear regression, with effect sizes reported for practical significance. A statistical significance level of *α* = 0.05 was adopted for all tests, with verification of statistical analysis assumptions before applying parametric methods.

## 8 Ethical considerations

This study received full ethical approval from the Research Ethics Committee at Imam Mohammad Ibn Saud Islamic University (IMSIU: 26443, 21/10/2025). All research procedures were conducted in accordance with the ethical standards stipulated in the Declaration of Helsinki and guidelines for research with human participants. Before participation, electronic informed consent was obtained from all participants via the first page of the electronic questionnaire. The consent information included a clear explanation of the study’s objectives, procedures, potential risks and benefits, the voluntary nature of participation, and the right to withdraw at any time without consequences, with an emphasis that all data were collected for research purposes only. Participants could not access the questionnaire until they had electronically confirmed their consent.

## 9 Findings

### 9.1 Preliminary and descriptive analyses

Before conducting the main analyses, Normality was assessed using the Skewness and Kurtosis indices, which fell within the acceptable range of ±2. Multivariate outliers were examined and eliminated using the Mahalanobis distance criterion (*p* < 0.001).

To address Research Question 1 and test H1, the levels of AI application usage patterns, academic resilience, and burnout among graduate students in special education departments at Saudi universities. Subsequently, descriptive statistics were calculated for all study variables, and the levels of each variable were categorized as low, medium, or high based on a tripartite division of the theoretical range of the scale.

Variable levels were categorized using a tripartite division of the theoretical scale range. For the AI Usage Scale (20 items, range 20–100): Low = 20–46.67, Medium = 46.68–73.33, High = 73.34–100. For the BRS (6 items, range 6–30): Low = 6–14, Medium = 14.01–22, High = 22.01–30. For the MBI-SS Total (10 items, range 0–60): Low = 0–20, Medium = 20.01–40, High = 40.01–60. This approach is consistent with prior Saudi studies employing the same instruments (Siraj et al., 2022). Table 3 presents these results.

**Table 3:** Descriptive statistics and Pearson correlation matrix for study variables (*N* = 367).

Variable	*M*	SD	1	2	3	4	5
1. AI: cog. & tech. proficiency	11.49	3.82	—				
2. AI: academic application	13.26	3.24	0.594**	—			
3. AI: spec. and adaptation	12.66	3.19	0.564**	0.789**	—		
4. Academic resilience	10.55	2.90	0.726**	0.687**	0.869**	—	
5. Burnout (total)	31.67	6.06	−0.395**	−0.541**	−0.562**	−0.437**	—

*M* and SD represent the mean and standard deviation, respectively.

**p < 0.01 (*N* = 367).

The findings indicate that the overall level of AI application use among graduate students was low. Similarly, students demonstrated a low level of academic resilience. In contrast, the results revealed a high level in the total burnout score, primarily attributed to high levels in the dimensions of emotional exhaustion and depersonalization, and a very low level in the personal accomplishment dimension.

### 9.2 Correlational relationships

To examine Research Question 2 and test H2, the relationships between patterns of AI application use, academic resilience, and burnout among graduate students in special education departments at Saudi universities were examined using a Pearson correlation matrix, as shown in Table 4.

**Table 4:** Pearson correlation matrix for the main study variables.

Variable	1	2	3
1. AI application usage patterns (total)	—		
2. Academic resilience	0.870**	—	
3. Burnout (total)	−0.541***	−0.437***	—

**p < 0.01; ***p < 0.001.

As shown in Table 4, the results indicated a statistically significant, moderate-strength negative correlation between the total score for AI application usage patterns and the total score for burnout (*r* = −0.541, *p* < 0.001). A statistically significant, moderate-strength negative correlation was also found between academic resilience and burnout (*r* = −0.437, *p* < 0.001).

### 9.3 Predictive power for burnout

To answer Research Question 3 and test H3, addressing whether AI usage patterns and academic resilience significantly predict burnout, two linear regression models were conducted.

### 9.4 Model 1: predicting burnout from AI application usage patterns

A standard multiple regression analysis was performed to assess the ability of the dimensions of AI use to predict burnout. The results, presented in Table 5, showed that the statistical model was significant, *F*(2, 364) = 94.29, *p* < 0.001, and explained 34.1% of the variance in burnout scores (*R*^2^ = 0.341). The analysis revealed that when the three dimensions were entered together, “Academic and Research Application” (*β* = −0.358, *p* < 0.001) and “Specialization and Adaptation” (*β* = −0.259, *p* < 0.001) were significant negative predictors of burnout, whereas “Cognitive and Technical Proficiency” did not contribute uniquely and significantly to the prediction, likely due to its shared variance with the other two dimensions.

**Table 5:** Results of multiple regression analysis for predicting burnout from AI application usage patterns.

Predictor	*B*	SE *B*	*β*	*t*
(Constant)	31.549	0.929		33.974***
Academic and research application	−0.560	0.108	−0.358	−5.172***
Specialization and adaptation	−0.397	0.106	−0.259	−3.737***
*R*	0.584			
*R* ^2^	0.341			
*F*	94.29***			

The dependent variable is the total score for burnout; ***p < 0.001.

In addition to statistical significance, the effect size was calculated to estimate the practical importance of the predictive model. Using Cohen’s *f*^2^ formula (*f*^2^ = *R*^2^/(1 – *R*^2^)), the effect size for the model was *f*^2^ = 0.52, which is classified as a large effect size according to Cohen (1988) criteria (*f*^2^ > 0.35). This indicates that AI application usage patterns not only have statistical significance but also substantial practical importance in predicting burnout levels among graduate students.

### 9.5 Model 2: predicting burnout from academic resilience

A simple linear regression analysis was conducted to assess the ability of academic resilience to predict burnout. As shown in Table 6, the statistical model was significant, *F*(1, 365) = 86.11, *p* < 0.001. Academic resilience explained 19.1% of the variance in burnout (*R*^2^ = 0.191). The results showed that academic resilience was a statistically significant negative predictor of burnout (*β* = −0.437, *p* < 0.001).

**Table 6:** Results of simple linear regression for predicting burnout from academic resilience.

Predictor	*B*	SE *B*	*β*	*t*
(Constant)	27.114	0.885		30.621***
Academic resilience	−0.751	0.081	−0.437	−9.280***
*R*	0.437			
*R* ^2^	0.191			
*F*	86.11***			

The dependent variable is the total score for burnout; ***p < 0.001.

**Figure 2:**
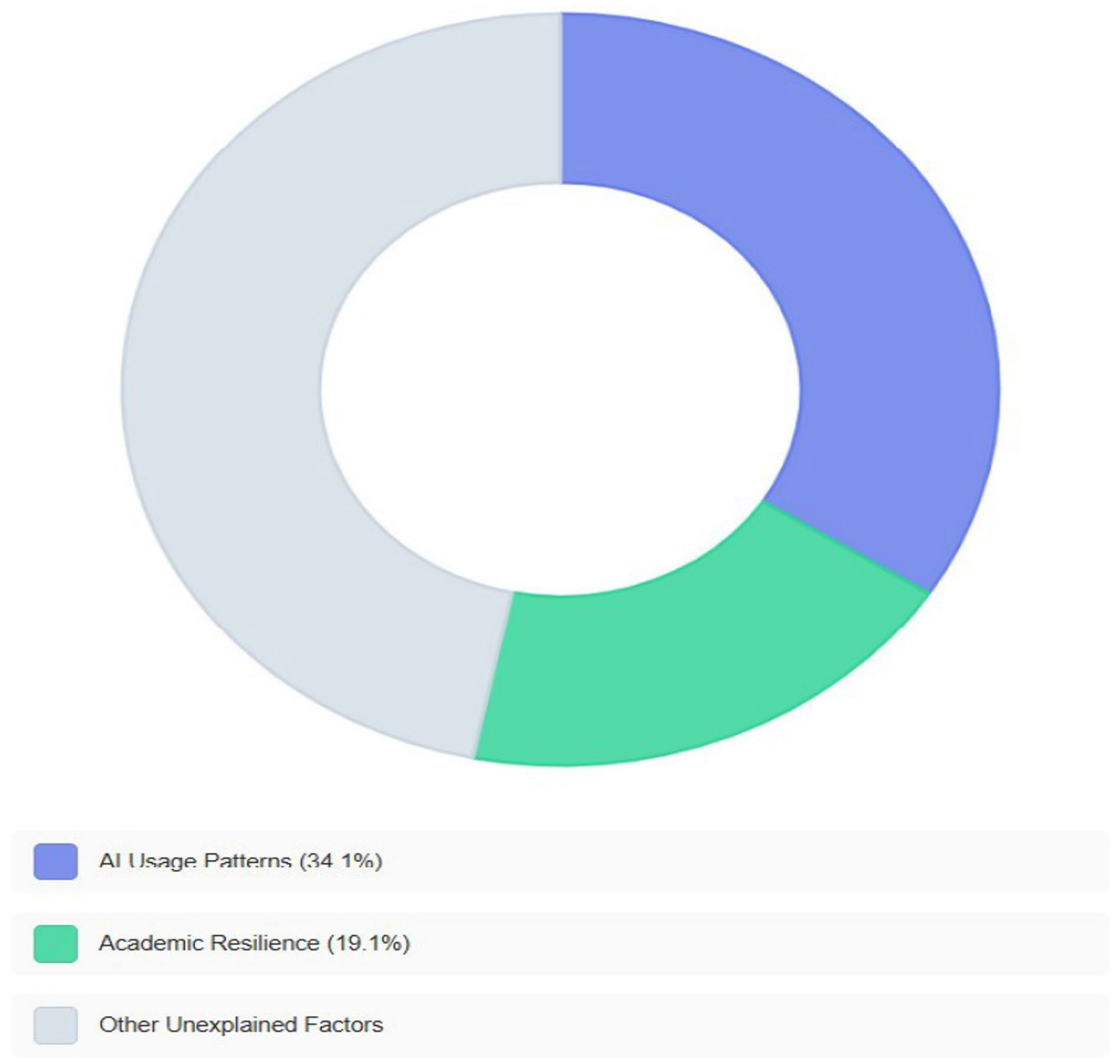
Proportion of variance in burnout explained by the predictor variables.

To evaluate the practical importance of this model, the effect size was calculated (*f*^2^ = *R*^2^/(1 – *R*^2^)). The result, *f*^2^ = 0.24, is classified as a medium to large effect size according to Cohen (1988) criteria (where *f*^2^ between 0.15 and 0.35 represents a medium effect). Although the predictive power of academic resilience (19.1%) is less than that of AI usage patterns (34.1%), it remains a tangible and practically important predictor. Figure 2 shows that AI application usage patterns explain 34.1% of the variance, academic resilience explains 19.1%, and other unmeasured factors account for the rest.

## 10 Discussion

The results of the current study revealed that the research sample of graduate students in special education departments at participating Saudi universities reported high levels of burnout, manifested primarily in the dimensions of emotional exhaustion and depersonalization. This coincided with a relatively low levels in the levels of AI application use in academic and research contexts, as well as in academic resilience scores. These descriptive findings are consistent with the theoretical expectations derived from the Job Demands-Resources (JD-R) model, which posits that burnout arises when academic and research demands exceed the student’s available resources (Asfahani, 2024; Siraj et al., 2023). In the context of graduate studies in special education departments, where research and academic pressures are intensifying, it appears that the low level of supportive resources—whether technological, represented by the limited use of smart applications, or psychological, represented by weak academic resilience—has contributed to the rise in emotional exhaustion and depersonalization, the two most prominent dimensions of the burnout syndrome in this cohort. These findings align with previous local studies conducted in the Saudi context on students in health specializations, which found high rates of burnout among students exposed to similar academic pressures (Alshobaili et al., 2021; Dabbagh et al., 2023).

While the current findings are consistent with broader burnout literature, it is important to note that academic burnout, as measured by the MBI-SS, is conceptually distinct from occupational burnout: its antecedents are rooted in academic demands (coursework, research pressures) rather than workplace stressors, and its consequences are primarily manifested in academic disengagement and reduced scholarly productivity (Schaufeli et al., 2002). The low levels of AI application use among the study sample can be interpreted through several interrelated mechanisms: First, this finding may reflect a knowledge gap in how to ethically and effectively employ these technologies within the academic research context, especially given the novelty of these tools and the limited systematic training programs available to students. Second, institutional and individual concerns about academic integrity—as indicated by studies from Aldawsari and Almohish (2024) and Hurshman et al. (2025)—may lead to student reluctance to use these applications for fear of being accused of plagiarism or a lack of research originality, even in cases where their use is legitimate and supportive. Third, limitations related to technological infrastructure (limited access to advanced paid versions, and internet connectivity disruptions in some contexts) may restrict the ability to fully utilize these tools. Fourth, this may also reflect cultural attitudes that favor traditional methods in academic research or a lack of trust in the accuracy and appropriateness of AI-generated outputs for the nature of humanities and educational specializations like special education. This multidimensional interpretation is supported by the study of Alotaibi and Alshehri (2023), which pointed to a gap between national digital transformation policies and actual practices at the institutional and individual levels.

In the same vein, the decline in academic resilience can be interpreted as a reflection of the cumulative psychological and academic pressures faced by students in the absence of adequate support. Resilience, as literature has shown, is not a fixed innate trait but a dynamic process that can be developed and enhanced (Abdelrahman et al., 2025; Wadi et al., 2024). When the academic environment lacks supportive resources—whether technological, psychological, or institutional—students’ ability to recover quickly from academic setbacks and persevere in the face of challenges diminishes. This is consistent with previous local studies that have confirmed that high-pressure academic environments, especially in high-demand specializations, are associated with decreased resilience levels among students (Almurdi et al., 2024; Al-Zain and Abdulsalam, 2022). Consistent with the JD-R framework, these results indicate that when AI tools function as effective resources—supplementing students’ capacity to meet research demands—they reduce the demand-resource imbalance that underlies burnout development. Conversely, limited AI utilization may amplify perceived demands, leaving the resource pool insufficient to buffer emotional exhaustion.

Furthermore, the correlation analysis revealed a statistically significant, moderate-strength negative relationship between the total score for AI application usage patterns and the total score for burnout (*r* = −0.541, *p* < 0.001). This suggests that students who make greater use of AI applications in their academic and research tasks tend to report lower levels of burnout. This finding can be interpreted within the JD-R framework, where AI applications act as a supportive technological resource that alleviates the cognitive and time-related burdens associated with complex research tasks, such as summarizing literature and formulating research ideas (Alotaibi, 2025; Cheng et al., 2025). When students can effectively employ these tools, they can manage academic demands with greater efficiency, which reduces emotional exhaustion and enhances their sense of personal accomplishment (Alazemi et al., 2024). Specifically, proficiency in “Academic and Research Application” functions as a precise buffer against “Emotional Exhaustion” and “Depersonalization”, confirming that targeted AI skills directly alleviate the core dimensions of academic burnout.

The potential risks of excessive digital reliance—including what the literature terms “technostress” (Lițan, 2025; Tarafdar et al., 2019) or “digital cognitive overload” (Stănescu and Romașcanu, 2024)—represent a competing mechanism that warrants investigation, particularly when AI use is unregulated or compulsive rather than purposeful. However, this result differs from expectations that might assume excessive use of digital technologies could increase digital stress and contribute to burnout (Hurshman et al., 2025). This discrepancy may be attributed to the nature of the usage itself; it appears that the structured and purposeful use of these applications—as measured by the dimensions of the scale used in this study, which focuses on cognitive proficiency and ethical academic application—differs fundamentally from random or addictive use, which some studies have warned against (Aldawsari and Almohish, 2024). This highlights the importance of distinguishing between different patterns of use when assessing the impact of smart technologies on students’ psychological well-being.

The findings also revealed a statistically significant, moderate-strength negative correlation between academic resilience and burnout (*r* = −0.437, *p* < 0.001), which is entirely consistent with theoretical and empirical literature confirming the protective role of resilience against burnout. Previous studies conducted in the Saudi context have indicated that resilience acts as a protective factor that reduces emotional exhaustion and helps students recover from academic pressures (Alsharif, 2020; Altharman et al., 2023). This relationship can be explained through theoretical models which suggest that individuals with higher levels of resilience possess better coping skills, such as self-regulation, positive thinking, and perseverance, enabling them to deal effectively with intense academic demands without falling into a state of emotional depletion (Abood and Hmaid, 2023; Al-Zain and Abdulsalam, 2022). From an educational psychology perspective, these findings underscore the role of technological self-efficacy and resilience as crucial self-regulatory mechanisms that sustain student motivation and mental health under pressure.

However, the strength of the relationship in the current study (*r* = −0.437) is considered moderate, suggesting the potential influence of additional contextual factors that were not directly measured, such as the level of institutional support or certain social and cultural aspects. This moderate relationship highlights the uniqueness of the Saudi cultural context, where resilience is often heavily intertwined with strong family support systems and societal expectations. Previous studies on Saudi populations, albeit in medical contexts (Al-Awad, 2024; Alsharif, 2020), have similarly demonstrated that resilience operates as a cultural buffer against systemic pressures. Previous studies have indicated that societal pressures and high family expectations—especially for female students—may reduce the effectiveness of resilience as a standalone protective factor (Alshobaili et al., 2021; Al-Awad, 2024). These findings highlight the need for multivariate analytical models that take into account the interaction of psychological, technological, and contextual factors.

Furthermore, the findings revealed a very strong, statistically significant positive correlation between AI application usage patterns and academic resilience (*r* = 0.870, *p* < 0.001). This reflects the continued impact of the effective use of smart technologies on academic performance, as it contributes to enhancing students’ ability to adapt to challenges and persevere in the face of difficulties. This can be explained by the fact that smart technologies, when used thoughtfully, provide tools that help students manage complex research tasks more efficiently, including information gathering, literature summarization, and improving the quality of research writing. This increases their sense of self-efficacy and control over the learning process, which is the core of academic resilience (Abdelrahman et al., 2025; Almankory, 2025; Alotaibi, 2025).

However, this exceptional relationship (*r* = 0.870) requires a degree of caution in interpretation; it may reflect a conceptual overlap between the two variables or the influence of common mediating factors that were not fully controlled for. It is important for future studies to explore the mechanisms that link the use of smart technologies and academic resilience through longitudinal or experimental designs that allow for tracking the temporal development of the variables and examining causal relationships more accurately.

The multiple regression analysis also showed that the Academic and Research Application and Specialization and Adaptation dimensions—rather than general Cognitive and Technical Proficiency—that emerged as significant predictors, suggesting that the protective effect of AI application usage patterns is contingent upon purposeful, domain-specific application rather than basic technological familiarity. The statistical model explained 34.1% of the variance in burnout scores. This result goes beyond merely establishing a correlational relationship, as it suggests that the structured and purposeful use of these applications in the academic context can actively contribute to reducing the risk of burnout. This can be explained within the JD-R model, where these applications act as supportive technological resources that increase students’ efficiency in managing research demands, thereby reducing emotional depletion and enhancing their sense of accomplishment (Asfahani, 2024). Aligned squarely with the JD-R model, AI proficiency and academic resilience operate as vital personal and technological “resources” that successfully counterbalance excessive research “demands”, preventing the trajectory toward emotional exhaustion.

This finding is particularly significant in the Saudi context, as it indicates that the integration of smart technologies in higher education—as targeted by Vision 2030—can have tangible positive effects on students’ psychological well-being, provided that this integration is done in a thoughtful and structured manner (Alotaibi and Alshehri, 2023; Khan et al., 2025). It is noteworthy that the cognitive and technical proficiency dimension did not contribute uniquely and significantly to predicting burnout when entered with the other two dimensions. This suggests that merely possessing basic technical skills is not sufficient on its own to achieve a protective effect; rather, the actual and specialized application of these technologies in academic and research tasks is the decisive factor. This is consistent with previous studies that have indicated that the quality and context of use are more important than its quantity or mere technical familiarity (Alotaibi, 2025; Cheng et al., 2025).

Regarding academic resilience, the simple linear regression analysis showed that it is a statistically significant negative predictor of burnout, explaining 19.1% of the variance in its scores. Although this percentage is lower than that explained by the model of usage patterns, it confirms the crucial role of resilience as a protective factor against burnout, as asserted by previous literature (Alsharif, 2020; Altharman et al., 2023). However, this finding raises important questions about other factors that may contribute to explaining the remaining portion of the variance (more than 80%), suggesting the need to incorporate additional variables in future predictive models, such as the level of institutional and social support, family circumstances, and other personal factors.

This result aligns with previous studies in the Saudi context, which have indicated that resilience plays a protective role against emotional exhaustion but may not be sufficient on its own to fully predict burnout in the absence of other supportive resources (Alsharif et al., 2023; Al-Zain and Abdulsalam, 2022). This reinforces the need to adopt integrated analytical models that consider the complex interactions between psychological, technological, and contextual variables. For example, the interaction between academic resilience and AI application usage patterns may have a synergistic effect that surpasses the impact of each variable separately, which requires a more detailed statistical examination in future studies.

It is also important to note that the current findings challenge some common assumptions about the potential negative effects of using smart technologies. Instead of AI application usage patterns being a risk factor that increases burnout—as might be assumed in light of concerns about excessive digital dependency or threats to academic integrity (Hurshman et al., 2025)—the findings have shown that the structured and ethical use of these applications can be a protective factor that enhances psychological well-being. This underscores the importance of focusing on how these technologies are used, not just their presence or absence, and highlights the need for adequate training and guidance for students to ensure they are employed effectively and responsibly. Such an interpretation is supported by recent literature emphasizing that ethical awareness and the responsible use of generative AI are essential for fostering meaningful digital engagement in educational settings (Sallam and Sallam, 2025).

## 11 Contributions and recommendations

### 11.1 Scientific and theoretical contributions

This study addresses a clear research gap by examining graduate students in special education in the Saudi context, after previous research had been confined to undergraduate students in health specializations. The study expands the JD-R model by incorporating a contemporary technological variable (AI application usage patterns) as a potential academic resource, offering an analytical framework that links technology, resilience, and burnout within an integrated model.

The empirical findings challenge common assumptions about the potential risks of smart technologies, showing that structured and ethical use acts as a protective factor, not a risk factor. The study has also developed a new scale for measuring patterns of AI application use with acceptable psychometric properties, providing a measurement tool for future studies and enriching the discussion on enhancing the psychological well-being of graduate students within the national orientations towards digital transformation.

A key methodological contribution of this study is the development and preliminary validation of the AI Usage Patterns Scale—a 20-item, three-dimensional instrument tailored to the graduate academic context in Saudi Arabia. This scale addresses a recognized measurement gap in the Arabic literature and provides a replicable, culturally sensitive tool for assessing AI adoption in higher education research.

From an educational psychology perspective, these findings extend the JD-R model to the technology-mediated learning context, suggesting that AI tools constitute a novel category of psychological resources. This has implications for resilience-based interventions in educational settings, proposing that technological competence training may function as a complementary route to traditional resilience-building programs.

### 11.2 Practical and applied recommendations

Given that the Academic and Research Application dimension was the strongest predictor of burnout (*β* = −0.358), training programs should prioritize applied AI skills—such as literature synthesis, writing assistance, and idea formulation—over general technical orientation. Similarly, given that resilience explained 19.1% of burnout variance, institutional resilience-building workshops represent a complementary, evidence-based intervention.

The research requirements of graduate programs should be restructured to achieve an optimal balance between demands and available resources, and systems for the early detection of burnout with preventive intervention mechanisms should be developed. The academic environment also requires an updated technological infrastructure and clear policies that regulate the use of AI in a way that preserves academic integrity. Faculty members are encouraged to adopt a supportive approach that provides constructive feedback, and to create learning communities that allow students to exchange experiences and mutual support.

These interventions align with Saudi Vision 2030 by enhancing researchers’ digital competence and academic resilience, while supporting digital transformation and a knowledge-based economy. Integrating AI literacy into graduate programs further advances Quality of Life objectives and national human capital development goals.

## 12 Limitations and future directions

Despite the scientific and practical contributions of this study, it is subject to a number of methodological limitations that should be considered when interpreting and generalizing its findings. The cross-sectional design limits the ability to draw causal conclusions; the observed correlational relationships do not prove direct causality but may reflect mediating variables that were not measured. The reliance on self-report instruments exposes the results to socially desirable response biases, while the convenience sample limits the representativeness of the findings and their generalizability. Furthermore, the sample is strictly confined to graduate students in special education departments, which restricts the generalizability of the findings to other humanities or applied science disciplines. For the “AI application usage patterns Scale,” face validity and internal consistency were verified. Given the novelty of the scale and the sample size, it is recommended to conduct factor analyses (EFA/CFA) in future studies to enhance the evidence of its construct validity.

The study did not address important contextual variables such as family support, economic status, and the nature of the relationship with the academic advisor, which may influence the studied relationships. The measurement of burnout as a general construct without detailed differentiation may hide important variations. The developed scale needs further verification across diverse contexts. The study’s focus on the Saudi context limits its generalizability to other cultural settings. These limitations call for conducting longitudinal studies to examine causal relationships across program stages, and experimental studies to test the effectiveness of specific interventions. Mixed-methods approaches should be used to provide a deeper understanding of the lived experiences, and samples should be expanded to include diverse specializations and regions. It is also recommended to examine mediating and moderating variables, conduct cross-cultural comparative studies, and develop more precise measurement tools that distinguish between different types of AI use.

Future research should also examine the long-term effects on research and critical thinking skills, explore factors that facilitate or hinder the effective use of these technologies, and use advanced analytical methods such as structural equation modeling to uncover more nuanced patterns of relationships between variables.

Future research should employ longitudinal designs to track within-individual changes in AI usage, resilience, and burnout trajectories across the graduate program lifecycle. Additionally, randomized controlled trials examining the efficacy of structured AI training interventions on burnout reduction would provide causal evidence that the present cross-sectional design cannot supply.

## 13 Conclusion

The findings of this study indicate that graduate students in special education departments at Saudi universities are facing high levels of burnout, manifested in emotional exhaustion and depersonalization, along with relatively low levels of use of AI applications and academic resilience. The strong negative correlations suggest that these two variables act as potential protective factors against the deterioration of psychological well-being.

The findings are particularly significant according to the Job Demands-Resources model, where sufficient technological and psychological resources are linked to lower levels of emotional depletion among students, suggesting their potential protective role in mitigating the effects of chronic academic stress. The statistically significant predictive ability of both variables indicates that they are important entry points for preventive and therapeutic interventions.

However, the methodological limitations call for caution in interpretation, and the unexplained variance in the regression models points to the existence of additional factors shaping the burnout experience. The study provides a basis for developing institutional policies and intervention programs that responsibly integrate smart technologies, develop coping skills, and restructure the academic environment to achieve a better balance. Within the context of Saudi Vision 2030, these findings acquire strategic importance related to the quality of educational outcomes and the sustainability of human capital.

Ultimately, this study provides preliminary yet substantive evidence that the thoughtful integration of AI tools and the cultivation of academic resilience represent complementary pathways toward healthier, more sustainable graduate education in Saudi Arabia—a contribution that speaks both to the imperatives of Vision 2030 and to the enduring mission of supporting human flourishing within higher education.

## Data Availability

The raw data supporting the conclusions of this article will be made available by the authors, without undue reservation.
