# Correction: The relationship between patterns of artificial intelligence use, academic resilience, and burnout among graduate students in special education departments at Saudi universities

**DOI:** 10.3389/fpsyg.2026.1898628

**Published:** 2026-06-22

**Authors:** Reda Ebrahim Mohamed Elashram, Liyla Marzouk Alamri

**Affiliations:** Department of Special Education, College of Education, Imam Mohammad Ibn Saud Islamic University (IMSIU), Riyadh, Saudi Arabia

**Keywords:** academic resilience, artificial intelligence applications, burnout, graduate students, Saudi universities, special education

In the published article, affiliation Department of Special Education, College of Education, Imam Mohammad Ibn Saud Islamic University (IMSIU), Riyadh, Saudi Arabia was erroneously given as Special Education Department, College of Education, Imam Mohammed Ibn Saud Islamic University, Riyadh, Saudi Arabia.

In addition, the **Funding** statement was omitted. The correct **Funding** statement is as follows: The author(s) declared that financial support was received for this work and/or its publication. This work was supported and funded by the Deanship of Scientific Research at Imam Mohammad Ibn Saud Islamic University (IMSIU) (grant number IMSIU-DDRSP2601).

The original version of this article has been updated.

